# Prevalence and predictors of confirmed infection in patients receiving empiric antimicrobials in the intensive care unit: a retrospective cohort study

**DOI:** 10.1016/j.bjane.2024.844567

**Published:** 2024-10-24

**Authors:** Luis Carlos Maia Cardozo Júnior, Larissa Bianchini, Jakeline Neves Giovanetti, Luiz Marcelo Almeida de Araújo, Yuri de Albuquerque Pessoa dos Santos, Bruno Adler Maccagnan Pinheiro Besen, Marcelo Park

**Affiliations:** a Faculdade de Medicina da Universidade de São Paulo, Hospital das Clínicas, Departamento de Emergência, Unidade de Terapia Intensiva, São Paulo, SP, Brazil; b Hospital Samaritano Paulista, Unidade de Terapia Intensiva, São Paulo, SP, Brazil; c Hospital A.C. Camargo Cancer Center, Unidade de Terapia Intensiva, São Paulo, SP, Brazil

**Keywords:** Anti-bacterial agents, Antimicrobial stewardship, Intensive care units, Healthcare associated infections

## Abstract

**Background:**

Infection diagnosis in Intensive Care Units (ICUs) is a challenge given the spectrum of conditions that present with systemic inflammation, the illness severity and the delay and imprecision of existing diagnostic methods. We hence sought to analyze the prevalence and predictors of confirmed infection after empirical antimicrobials during ICU stay.

**Methods:**

retrospective cohort of prospectively collected ICU data in an academic tertiary hospital in São Paulo, Brazil. We included all adult patients given a new empirical antimicrobial during their ICU stay. We excluded patients using prophylactic or microbiologically guided antimicrobials. Primary outcome was infection status, defined as confirmed, probable, possible, or discarded. In a multivariable analysis, we explored variables associated with confirmed infection.

**Results:**

After screening 1721 patients admitted to the ICU from November 2017 to November 2022, we identified 398 new antimicrobial prescriptions in 341 patients. After exclusions, 243 antimicrobial prescriptions for 206 patients were included. Infection was classified as confirmed in 61 (25.1%) prescriptions, probable in 39 (16.0%), possible in 103 (42.4%), and discarded in 40 (16.5%). The only factor associated with infection was deltaSOFA (OR = 1.18, 95% CI 1.02 to 1.36, p = 0.022).

**Conclusion:**

Suspected infection in the ICU is frequently not confirmed. Clinicians should be aware of the need to avoid premature closure and revise diagnosis after microbiological results. Development and implementation of new tools for faster infection diagnosis and guiding of antimicrobial prescription should be a research priority.

## Introduction

Sepsis and septic shock are common causes of Intensive Care Unit (ICU) admission and account for approximately 11 million deaths worldwide annually.^1^ During hospitalization, clinical worsening often raises the suspicion of a new infectious event or a previous infection not properly treated, and due to morbidity and mortality, rapid diagnosis and treatment have become indicative of good medical practice, resulting in a probable reduction in the number of deaths.^2^, ^3^, ^4^

However, diagnosis of infection in ICUs is a major challenge for the intensive care physician, given the wide spectrum of conditions that present as a systemic inflammatory response, the severity of the patients and the delay and imprecision of existing diagnostic methods.^5^ In this context, antimicrobials are administered to approximately 70% of individuals cared for in intensive care units, but approximately half of these prescriptions may be unnecessary.^6^^,^^7^

Both undertreatment and unnecessary antimicrobial prescriptions are associated with worse outcomes in patients with suspected sepsis.^8^ Excessive use of antimicrobials has been associated with significant increases in the occurrence of multidrug resistant microorganisms beyond the creation of new drugs. In addition, health services are burdened due to the costs of new drugs as well as due to side effects of the therapy.^6^

Previous research in emergency departments or with recently admitted ICU patients has shown that a sizable proportion of patients initially treated with antimicrobials ultimately have no infection or only possible infection.^9^^,^^10^ However, there are scarce data in the literature regarding prevalence of confirmed infection among patients admitted for other reasons in the ICU that developed signs or symptoms of infection, and received empirical antimicrobials during ICU stay. Considering the complex course of critical illness, with several confounders that may be sepsis mimics, results of previous studies may not apply to these patients. To fulfill this gap in the literature we sought to analyze, in a cohort of critically ill patients, the prevalence of confirmed infection after empirical antimicrobial prescription during ICU stay.

## Methods

### Study design and ethics

This is a retrospective cohort study taking advantage of a prospectively collected ICU database, reported in accordance with STROBE guidance.^11^ We followed all the recommendations of the Helsinki Declaration. The Research Ethics Committee approved the study protocol (number 6.122.265), and informed consent was waived.

### Setting

The study was conducted at a mixed/medical ICU of an academic tertiary hospital in São Paulo, Brazil. This ICU currently has 8 beds, however, the number of beds fluctuated from five to 12 during the study period depending on funding for staffing.

In this ICU there is a well-established antimicrobial stewardship program focused on appropriate antimicrobial use. In short, whenever a patient's condition worsens and there is suspicion of infection, there is a great effort to identify the infectious source (through laboratory tests, images, and cultures), as well as to identify diagnostic alternatives. If the patient is stable and without shock, antimicrobials are withheld, and the patient is observed until infection is confirmed or another cause for clinical worsening is identified. In case of deterioration or shock, empirical antimicrobials are started with the narrowest possible coverage (according to site of suspected infection and multidrug resistance pathogen risk factors). When an antimicrobial is started, its maintenance is re-evaluated daily and, facing early clinical improvement (< 12-hours), or late non-improvement (> 72-hours), mainly if cultures were negative and images non-suggestive of infection, the antimicrobial is withdrawn.^2^^,^^12^

### Participants

We assessed the ICU database, which is hosted in Microsoft Access® by the senior consultants in charge of the ICU, and includes baseline and daily data of all patients, used for administrative and benchmarking purposes. Through this database, we identified all adult patients (> 18-years) from November 2017 to November 2022 who started a new antimicrobial during their ICU stay. We excluded patients receiving antimicrobials in the first 48 hours after ICU admission to keep only patients with suspected nosocomial infections acquired in ICU.

The patients identified in screening had their electronic medical records reviewed to extract relevant data and ascertain infection status. The first step was to confirm that the new antimicrobial was started empirically. We excluded cases with prophylactic antimicrobials or prescription guided by microbiological confirmation (in which cultures were collected and antimicrobials were initially withheld, being started only after infection had been confirmed by cultures). We also excluded patients erroneously identified in the database (patients identified in initial screening that did not receive a new antimicrobial or that were already using an antimicrobial). Given that a patient could receive empirical antimicrobials several times during ICU stay, patients could be included more than once.

### Data collection and extraction

We extracted age, gender, SAPS3 (Simplified Acute Physiology Score III, measured in ICU admission), comorbidities (summarized by a modified Charlson comorbidity index, excluding age), immunosuppression status (high dose glucocorticoids, immunosuppressants, AIDS or chemotherapy), admission source (ward, emergency, operation room), antimicrobial use upon admission, initial diagnosis and status performance prior to ICU admission (assessed through ECOG scale – Eastern Cooperative Oncology Group performance status scale).^13^, ^14^, ^15^

Moreover, electronic medical records were reviewed and data regarding new antimicrobial start were collected, including time after hospital and ICU admission, Sequential Organ Failure Assessment (SOFA)^16^ on antimicrobial start day and variation in the previous 24h (deltaSOFA), mechanical ventilation and vasoactive drug use, vital signs, total leucocyte count, blood glucose levels on antimicrobial start day, and modified Systemic Inflammatory Response Syndrome (SIRS) criterion (see below). We also checked which antimicrobial was prescribed and results of microbiologic tests performed between 2 days before and 2 days after antimicrobial start. Finally, ICU and hospital length-of-stay, and mortality were registered.

### Outcomes and predictors

Our primary outcome was the proportion of confirmed infections among critically ill patients that received a new empirical antimicrobial during ICU stay. Since there is no practical gold standard for definition of confirmed infection in ICU, we adopted the definitions used by Hooper et al.,^9^ with some modifications to fit our database. Infections were defined as confirmed, probable, possible, or discarded (more details below).

Furthermore, we explored clinical and laboratory variables associated with confirmed infection. Predictors of infection were selected *a priori* based on clinical relevance for higher probability of infection, and included: SOFA on antimicrobial day, deltaSOFA, modified Charlson Comorbidity index, prior antimicrobial use upon admission, immunosuppression, mechanical ventilation or vasoactive drugs on antimicrobial day, maximum heart rate on antimicrobial day, maximum and minimum temperature on antimicrobial day, maximum blood glucose on antimicrobial day, and leucocytes on antimicrobial day.

### Definitions

We defined infections as follows: Confirmed: positive cultures of sterile sites (e.g., blood, cerebrospinal fluid) with use of antimicrobials for more than 48 hours. In case of potential contaminants (e.g., coagulase negative Staphylococci) we required growth in more than one sample and indication in medical records that antimicrobials were used to treat the microorganism. Probable: Positive cultures of non-sterile or potentially non-sterile sites (e.g., urine, tracheal aspirates) with use of antimicrobials for more than 48 hours. Possible: Negative cultures, but with a high enough suspicion to keep a full course of antimicrobials (more than 48–72 hours) or death in less than 48 hours of antimicrobial start (without another clear cause than infection). Discarded: None of the above (negative cultures and antimicrobials withdrawn within 48 hours). We considered that in critically ill patients with suspected nosocomial infection, a very short course of antimicrobials would potentially be insufficient for adequate treatment, so that improvement in this context was correlated with a very low probability of infection.

Both probable and possible categories were designed to reflect cases in which suspicion of infection was high enough to justify a full course of antimicrobials (or scenarios in which an infection could not be ruled out), but the main difference between them was that in cases classified as probable there was microbiological evidence of infection, while this was absent in cases assigned as possible infection. Moreover, the probable infection category addresses the intrinsic uncertainty regarding cultures of non-sterile sites (in which distinction between infection and colonization may be difficult). In possible infections, culture results were all negative, however patients were considered to have an infection, even with negative microbiological investigations, considering that in some scenarios culture yield is low. Culture harvesting was performed in accordance with the attending physicians whenever any infection was suspected. In general, at least two blood culture samples were obtained for all patients, with additional samples (e.g., tracheal aspirate, urine, catheter tip) collected according to the suspected source of infection.

To define the presence of SIRS we did not have the vital signs at the exact moment of decision to start an antimicrobial, but only the lower and the higher values in the day. To account for daily variation, we chose to modify some cut-offs to improve specificity. SIRS was defined by at least 2 of 4: leucocytes higher than 12,000 mm^3^ or lower than 4,000 mm^3^, temperature higher than 38°C or lower than 35°C, respiratory rate higher than 25 breaths per minute and heart rate higher than 110 beats per minute. In case of more than one leucocyte count on the same day we registered the highest.

After investigating the infectious status, we recorded the source of infection based on clinical and laboratory data. In case of an inconclusive source of infection, we assumed the source considered most likely as noted in the medical records. If it was still not possible to define the source, we considered it as unknown. Finally, to describe the microorganisms isolated in culture samples we used the same definition used in the Eurobact-2 cohort.^17^

### Statistical analysis

Categorical variables were expressed as numbers and percentages. We checked normality in the distribution of variables using histograms and the Shapiro test. As most variables had a non-normal distribution, quantitative variables are presented as median and 25–75^th^ percentile. Differences between categorical variables were assessed with chi-square or Fisher exact test, while the Kruskal-Wallis test was used for continuous variables. For the descriptive analysis of the primary outcome, binomial 95% Confidence Intervals are presented. Missing data were present in a small number of cases, so a complete case analysis was undertaken.

Given the observational nature of this study, we did not perform an *a priori* sample size estimation. We chose the five-year period for logistical reasons (related to the implementation of specific software for medical records in the hospital).

We assessed variables associated with higher odds of confirmed infection (predictors). In this analysis, infection status was dichotomized as present (confirmed, probable and possible) or absent (discarded). Considering that some patients were included in the study more than once (e.g., if a patient received 2 distinct empirical antimicrobial treatments along ICU stay, they could be included twice in the study), our observations in those cases were not independent, as some variables (e.g., age, immunosuppression, SAPSIII) were intrinsic to each patient. To overcome this, we used a hierarchical multiple logistic regression model accounting for clustering within patients in this analysis (by adding a term “patient id” in the model). Predictors were defined *a priori* (see above) and selection of variables to be retained in the final model was done with a backward stepwise elimination process using Akaike Information Criterion.

We planned some sensitivity analysis including: 1) Patients assigned “possible infection” because of death within 48h were considered “discarded”; 2) Infection status dichotomized as present (confirmed and probable – culture proven infection) and absent (possible and discarded); 3) An alternative model with numeric predictors dichotomized according to clinical practice (maximum heart rate higher than 120 beats per minute, maximum temperature higher than 38°C, minimum temperature lower than 35°C, leucocytes count higher than 15.000 cell.mm^−3^ and maximum glucose higher than 180 mg.dL^−1^). We also performed a *post-hoc* exploratory analysis to verify the number of patients with discarded infection who received another empiric antimicrobial course during their ICU stay and how many of them had infection confirmed thereafter. This *post-hoc* analysis was done to increase the certainty regarding absence of infection and ensure that classification done in the main analysis was accurate. All analyses were performed using *R* free source software^18^ version 4.1.3 and a p-value < 0.05 was considered for hypothesis testing.

## Results

### Descriptive analysis

**Figure 1:**
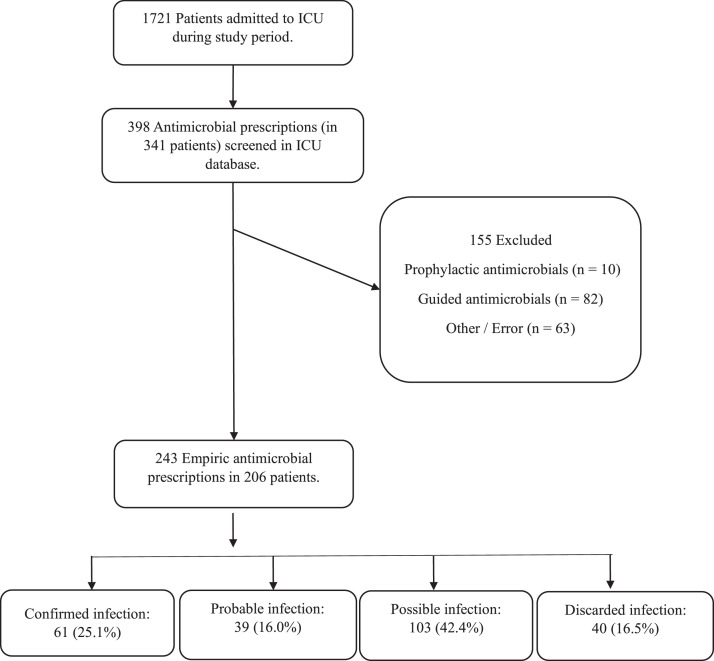
Study flowchart.

Between November 2017 and November 2022, a total of 1721 patients were admitted to the ICU study and included in the administrative database. After screening, we identified 398 new antimicrobial prescriptions in 341 patients. After exclusions, 243 antimicrobial prescriptions for 206 patients were included. A detailed description is presented in Figure 1. The general characteristics of the study patients are presented in Table 1. In summary, the cohort had high disease severity (median SAPS3 = 68.5) at ICU admission and at empirical antimicrobial initiation (median SOFA = 8), with high ICU (38%) and hospital (56%) mortality and long ICU and hospital stay. Most admissions came from the emergency department due to respiratory and neurological diseases.

**Table 1:** General characteristics of included patients.

Variables	N (%) or Median [P25, P75]
Age, years	55.00 [42.25, 66.75]
Sex: Male	121 (58.7)
SAPS3	68.50 [57.00, 80.75]
Modified Charlson	1.00 [0.00, 3.00]
Comorbidities	
*Hypertension*	108 (52.4)
*Uncomplicated diabetes*	17 (8.3)
*Complicated diabetes*	40 (19.4)
*Heart failure*	15 (7.3)
*COPD*	7 (3.4)
*Solid tumor*	10 (4.9)
*Cirrhosis*	4 (1.9)
ECOG	77 (37.4)
*0*	53 (25.7)
*1*	36 (17.5)
*2*	36 (17.5)
*3*	77 (37.4)
*4*	4 (1.9)
Antimicrobial use on ICU admission	149 (72.3)
Admission cause	
*Respiratory*	110 (53.4)
*Neurologic*	36 (17.5)
*Sepsis*	18 (8.7)
*Trauma*	10 (4.9)
*Gastrointestinal*	8 (3.9)
*Post-surgery*	8 (3.9)
*Cardiovascular*	6 (2.9)
*Other*	10 (4.9)
Admission source	
*Emergency department*	74 (35.9)
*Other ICU*	65 (31.6)
*Ward*	33 (16.0)
*Operation room*	33 (16.0)
*Other*	1 (0.5)
Immunosuppression	42 (20.4)
ICU length of stay	17.00 [10.00, 25.00]
Hospital length of stay	26.00 [16.00, 52.75]
ICU mortality	79 (38.3)
Hospital mortality	116 (56.3)

SAPS3, Simplified Acute Physiology Score III; COPD, Chronic Obstructive Pulmonary disease; ECOG, Eastern Cooperative Oncology Group performance status scale; ICU, Intensive Care Unit.

Infection was classified as confirmed in 61 (25.1%, 95% CI 20%–31%) prescriptions, probable in 39 (16.0%, 95% CI 12%–21%), possible in 103 (42.4%, 95% CI 36%–49%), and discarded in 40 (16.5%, 95% CI 12%–22%). Table 2 describes clinical variables present on the day of empirical antimicrobial prescription in the overall cohort and stratified by infection status. Data regarding antimicrobials used and pathogen characteristics are detailed in Table 3. There were no missing data regarding the primary outcome or patient characteristics. Regarding vital signs on the day the antimicrobial was initiated, we had very few missing values (one or two for most vital signs, except for SpO2 measurements, with eight missing values). Using a complete case analysis, these missing data led to the exclusion of only four antimicrobial prescriptions from the main model (which was run with 239 new antimicrobial prescriptions).

**Table 2:** Clinical variables on the day of empiric antimicrobial prescription, stratified by infection status.

Variable	Overall	Confirmed	Probable	Possible	Discarded
Number of prescriptions	243	61	39	103	40
Time after hospital admission, days	12.0 [8.0, 20.5]	13.0 [9.0, 22.0]	11.0 [7.0, 18.0]	12.0 [7.0, 21.0]	11.0 [7.7, 16.0]
Time after ICU admission, days	9.0 [6.0, 15.0]	10.0 [7.0, 15.0]	7.0 [4.5, 13.0]	8.0 [5.0, 14.5]	9.5 [6.0, 13.5]
SOFA score	8.0 [4.0, 13.0]	8.0 [3.0, 14.0]	9.0 [5.0, 13.0]	9.0 [5.0, 13.0]	7.0 [4.0, 11.0]
DeltaSOFA	1.0 [0.0, 4.0]	1.0 [0.0, 4.0]	1.0 [0.0, 3.0]	1.0 [0.0, 4.0]	0.0 [0.0, 2.2]
Mechanical ventilation, n (%)	155 (63.8)	38 (62.3)	30 (76.9)	61 (59.2)	26 (65.0)
Vasopressors, n (%)	105 (43.2)	28 (45.9)	19 (48.7)	45 (43.7)	13 (32.5)
Maximum heart rate, beats per minute	118.0 [104.0, 133.0]	121.0 [105.0, 135.0]	118.0 [107.0, 131.0]	115.5 [103.2, 130.7]	121.0 [99.5, 136.0]
Maximum temperature,°C	37.5 [37.0, 37.9]	37.4 [37.0, 37.8]	37.5 [37.1, 37.8]	37.5 [36.9, 37.9]	37.6 [36.9, 38.0]
Minimum temperature,°C	35.7 [35.1, 36.2]	35.6 [35.1, 36.1]	35.9 [35.5, 36.2]	35.6 [35.0, 36.1]	35.8 [35.2, 36.3]
Minimum oxygen saturation, %	86.0 [84.0, 90.0]	86.0 [82.5, 90.0]	86.0 [83.0, 90.0]	86.0 [85.0, 90.0]	88.0 [84.0, 91.0]
Total leucocytes count, cells.mm^−3^	18020 [11597.5, 25735.0]	17760 [11310.0, 24670.0]	20850 [15135.0, 26990.0]	18850 [12350.0, 25915.0]	14310 [10747.5, 21702.5]
Maximum blood glucose, mg.dL^−1^	156.0 [123.0, 220.0]	170.0 [124.7, 234.5]	166.0 [123.5, 261.5]	156.5 [124.2, 197.7]	148.0 [126.0,201.0]
Modified SIRS criteria, n (%)	199 (81.9)	49 (80.3)	33 (84.6)	83 (80.6)	34 (85.0)

ICU, Intensive Care Unit; SOFA, Sequential Organ Failure Assessment; deltaSOFA, Variation in SOFA score in the previous 24h before antimicrobial start; SIRS, Systemic Inflammatory Response Syndrome.

**Table 3:** Antimicrobials used and pathogen characteristics.

Variable	n (%)
Empiric antimicrobials used^a^	
*Vancomycin*	147 (60.5)
*Levofloxacin*	70 (28.8)
*Piperacillin + Tazobactam*	62 (25.5)
*Ciprofloxacin*	25 (10.3)
*Metronidazole*	13 (5.3)
*Ceftriaxone*	12 (4.9)
*Polymyxins*	10 (4.1)
*Clindamycin*	5 (2.0)
*Ampicillin*	4 (1.6)
*Ceftazidime*	4 (1.6)
*Amikacin*	3 (1.2)
*Fluconazole*	3 (1.2)
*Meropenem*	2 (0.8)
*Sulfamethoxazole + Trimethoprim*	2 (0.8)
*Other*	7 (2.8)
Empiric broad spectrum antimicrobials	170 (69.9)
Infection source	
*Respiratory*	112 (46.1)
*Abdominal*	23 (9.5)
*BSI*	21 (8.6)
*Skin / Soft tissue*	13 (5.3)
*Urinary*	8 (3.3)
*CNS*	7 (2.9)
*Endocarditis*	1 (0.4)
*Unknown*	18 (7.4)
*None (Infection discarded)*	40 (16.5)
Isolated pathogens	171
Gram positive bacteria	82 (48.0)
*Staphylococcus aureus / MRSA*	46 / 18
*Staphylococcus coagulase negative*^b^*/ MRSE*	21 / 12
*Enterococcus spp. / VRE*	8 / 0
*Other gram positives*	7
Gram negative bacteria	80 (46.8)
*Pseudomonas spp. / DTR*	28 / 1
*Klebsiella pneumoniae / DTR*	13 / 4
*Enterobacter spp. / DTR*	8 / 0
*E. coli / DTR*	6 / 0
*Acinetobacter spp. / DTR*	4 / 3
*Other gram negatives / DTR*	21 / 4
Fungi	7 (4.1)
*Candida albicans*	3
*Candida non-albicans spp.*	4
Strict Anaerobic bacteria	2 (1.2)

a Each prescription could include more than one antimicrobial, so total antimicrobial count is higher than the number of prescription events.

b Most coagulase negative staphylococci were considered contaminants. BSI, Bloodstream Infection; CNS, Central Nervous System; MRSA, Methicillin Resistant Staphylococcus Aureus; MRSE, Coagulase negative Staphylococcus Resistant to Methicillin; VRE, Vancomycin Resistant Enterococcus; DTR, Difficult to Treat Resistance. No pan-drug resistant bacteria were isolated.

### Association between predictors and infection

From the twelve predictors initially considered by clinical relevance, four variables were retained in the final model: deltaSOFA, antimicrobial use on ICU admission, mechanical ventilation, and total leucocyte count (Table 4). The only predictor with statistically significant association with infection was deltaSOFA (OR = 1.18, 95% CI 1.02 to 1.36, p = 0.0224).

**Table 4:** Variables associated with infection (confirmed, probable or possible).

Variables	Odds-Ratio	95% CI	p*-*value
DeltaSOFA	1.18	1.02 to 1.36	0.0224
ATB use upon ICU admission	1.69	0.79 to 3.57	0.170
Mechanical ventilation	0.56	0.25 to 1.26	0.162
Leucocytes (cells.mm^−3^/1000)	1.04	0.99 to 1.08	0.0587

CI, Confidence Interval; deltaSOFA, Variation in SOFA score in the previous 24h before antimicrobial start; ATB, Antimicrobial.

### Sensitivity analyses

If prescriptions in which patients died within 48h and cultures results were negative were assigned as discarded infection, 62 (25.5%) patients were considered to have absent infection. When the final model was run using this modified criterion, the association between deltaSOFA and infection was no longer present (Table S1). In another sensitivity analysis we considered that infection was present only if cultures were positive (confirmed and probable – culture proven infection). The hierarchical logistic regression model using this definition retained three variables: antimicrobial on admission, immunosuppression, and maximum serum glucose on antimicrobial day. Only immunosuppression had a statistically significant association with culture proven infection (Table S2). An alternative model with predictors dichotomized according to clinical practice is presented in Table S3.

In a *post-hoc* exploratory analysis we assessed the number of patients with discarded infection that received another empirical antimicrobial course during ICU stay. From 40 prescriptions classified as discarded, ten patients were prescribed another empirical antimicrobial after a median of 6 days after discarded infection (mean of 7.3 days). Among these ten patients, six were classified as possible, one as probable, and three as confirmed.

## Discussion

### Main findings

In this cohort of critically ill patients who received a new antimicrobial prescription for a suspected nosocomial infection, we observed that in 16.5% of the events an infectious condition was discarded and in more than 40% it was only possible (antimicrobials were maintained, despite negative cultures). Several predictors commonly used in clinical practice (e.g., leucocytes count, fever) failed to discriminate between events of infection present or discarded and the only predictor with a statistically significant association to infection was variation of SOFA score in the 24 hours before antimicrobial initiation.

### Relationship with the literature

There are few reports evaluating the incidence of nosocomial infections in patients with empirical antimicrobial use. Our results were similar to a previous observational study that evaluated a cohort of patients admitted to ICU with suspected infection and found that 43% of them had an infection status categorized as discarded or possible.^10^ Another more recent cohort including only patients treated in emergency department with suspected sepsis found lower incidences, with 9.5% of patients with infection discarded and 9.7% with only a possible infection.^9^ Nevertheless, our findings reflect an already restrictive antimicrobial prescription culture, in which empiric antimicrobials were started only in cases of shock or very high suspicion of infection. Possibly, false positive rates would be higher in other scenarios where a more liberal antimicrobial prescription policy is present.

Our criteria for defining infection were different from previous trials, which may explain some of the differences in our results. We relied on culture results and time on antimicrobials to define the presence of infection, and although this approach has some limitations, it made our infection status classification process more objective, reducing the risk of measurement bias. Furthermore, objective inclusion and exclusion criteria allowed patient selection before data collection began, reducing the risk of selection bias.

We aimed to evaluate predictors of confirmed infection by a hierarchical logistic regression model. While needed to overcome the clustering of observations between repeated patients, this model had some limitations, including a low number of patients, some imprecision in the estimates and possible non-linear relationships between outcomes and predictors. Despite these limitations, some observations caught our attention: 1) Common markers of infection used in clinical practice (temperature, leucocyte count, heart rate) were not able to discriminate between events of confirmed or discarded infection, reflecting low specificity in a population of critically ill patients with high disease severity and often multiple organ dysfunction; 2) In the main model, deltaSOFA was able to predict infection, but this was no longer true in sensitivity analysis (when we considered only patients with culture proven infection or used a different definition for possible infection assigning patients who died within 48 hours of antimicrobial prescription with negative cultures as discarded). In this scenario, deltaSOFA was possibly a marker of more severe disease (which led to maintenance of antimicrobials) and not a marker of infection itself.

This is not surprising, as the reasons for prescribing or continuing empirical antimicrobials may be related to perceived risks of withholding this therapy, constraints by local protocols or regulatory norms and concerns regarding ethical and legal consequences of undertreatment, and also are influenced by the degree of uncertainty and difficulty in reaching a correct diagnosis.^19^ In a previous report, antimicrobials were given even when conviction of an infection was low and it was confirmed only in 54% of the cases.^20^ Despite advocacy of most sepsis guidelines for early and aggressive antimicrobial treatment, evidence supporting this strategy is controversial.^3^^,^^7^^,^^21^, ^22^, ^23^ Both undertreatment and unnecessary antimicrobial use may be harmful and expose patients and health systems to undesirable consequences.^8^^,^^24^

### Implications for practice and research

Efforts should be made to increase precision and certainty regarding infection diagnosis in critically ill patients. This is a major challenge as there is no gold standard for infection diagnosis, clinical course and presentation is highly heterogenous, and several other conditions present in ICU may be sepsis mimics (e.g., atelectasis, chemical phlebitis, neurologic diseases, pulmonary embolism, drug reactions). Microbiologic cultures are the current reference for infection diagnosis; however, their results take time, may be influenced by sampling techniques and sensitivity may be low depending on the suspected source of infection. Moreover, factors like contamination and previous antimicrobial use may further affect culture accuracy.^5^

In this study, we found that change in SOFA score in the 24 hours prior to antimicrobial initiation was the only independent predictor of confirmed infection. Despite some concerns, this finding is novel and should undergo prospective validation in future trials before widespread use.

New tools such as Multiplex Polymerase Chain Reaction and chromogenetics are not widely available but may have a role in the future, allowing faster identification of an infectious agent and guiding of antimicrobial therapy.^25^^,^^26^ Machine learning algorithms able to use real time data to predict sepsis and septic shock in critically ill patients have emerged recently with promising results, however those methods still require prospective validation and standardization.^27^, ^28^, ^29^ Further research and implementation of more precise diagnostic tools for infection in critically ill patients should be a priority. Meanwhile, clinicians should be aware that a considerable number of patients exposed to empirical antimicrobials may not have an infection. Daily reassessment of infection status, including clinical course and microbiologic findings, is critical to identify situations in which antimicrobials can be safely withdrawn.

### Limitations

This study has several limitations. It was conducted in a single ICU in an academic hospital of a middle-income country, with selective antimicrobial practices, which reduces generalizability of the findings to other ICU settings. However, our results were in line with previous publications performed in North America and Europe. We excluded patients with suspected nosocomial infections acquired outside the ICU (e.g., in the ward) that led to admission to the intensive care unit and focused only on the patients that worsened during their ICU stay. We did not have granular data before ICU admission and it is important to consider that nosocomial infections acquired in the ICU may have a different clinical course compared to infections developed in the ward/emergency room, which would have introduced heterogeneity to our results. Confirmed and probable infections were defined based on positive cultures, which may be biased by several factors such as prior antibiotic use, presence of fastidious organisms, localized infections, presence of low-level bacteremia, or specific infections where culture yield is low. Moreover, we did not analyze detailed clinical or laboratory data to adjudicate the presence of infection. Our criterion for discarded infection was arbitrary and based on withdrawing of antimicrobials within 48 hours with clinical improvement. We believe that a very short antimicrobial course would be inappropriate for most ICU-acquired infections (specially in our cohort with high disease severity), but for some patients this short course could have been sufficient for improvement. The sample size was not large, but we had at least 50 events/non-events to perform analysis, enough for inclusion of at least 4 or 5 strong predictors. Nevertheless, only deltaSOFA was statistically associated with the primary outcome.

It is worth mentioning that the COVID-19 pandemic occurred in the middle of our study period and around 40% of included patients were admitted to the ICU with infection by SARS-CoV-2 as the primary diagnosis. Although our practices regarding antibiotic prescriptions did not change during the pandemic, we had more patients under mechanical ventilation, deep sedation and paralysis, which may expose patients to nosocomial infections. Furthermore, COVID-19 can cause inflammatory signs even weeks after the onset of infection, which may also raise suspicion of a secondary infection. Altogether, there is a chance that our results may have been influenced by the COVID-19 pandemic.

## Conclusions

Our data suggest that even in an ICU with a restrictive antimicrobial program, in a noteworthy part of antimicrobial prescriptions an infection was not confirmed. Clinicians should be aware of the need to avoid premature closure and revise diagnosis after microbiological results. Development and implementation of new tools for faster infection diagnosis and guiding of antimicrobial prescription should be a research priority.

## Funding

This research received no specific grant from any funding agency in the public, commercial, or not-for-profit sectors.

## Conflicts of interest

The authors declare no conflicts of interest.

## Appendix Supplementary materials

Image, application 1

## Footnotes

Supplementary material associated with this article can be found in the online version at doi:10.1016/j.bjane.2024.844567.
