# Supplementary material for: Prevalence and predictors of confirmed infection in patients receiving empiric antimicrobials in the intensive care unit: a retrospective cohort study
Source: Braz J Anesthesiol. 2024 Oct 24;75(1):844567. doi: 10.1016/j.bjane.2024.844567 (PMC11846440; doi:10.1016/j.bjane.2024.844567)
Supplement: Supplementary file 1 [file mmc1.docx]

**BJAN-D-24-00155_ Supplementary Material**

**Supplemental Material**

**Table S1** Predictors associated with infection present (confirmed, probable or possible) using a modified criterion for possible and discarded infection (patients that died in 48h after antimicrobial start and had negative cultures were considered “discarded”).

| **Variables** | **Odds-Ratio** | **95% CI** | **p-value** |
| --- | --- | --- | --- |
| DeltaSOFA | 0.96 | 0.88 to 1.06 | 0.494 |
| ATB use upon ICU admission | 1.34 | 0.69 to 2.56 | 0.384 |
| Mechanical ventilation | 0.82 | 0.41 to 1.66 | 0.590 |
| Leucocytes (cells.mm^-3^/1000) | 1.01 | 0.98 to 1.05 | 0.344 |

CI, Confidence Interval; deltaSOFA, Variation in SOFA score in the previous 24h before antimicrobial start; ATB, Antimicrobial.

**Table S2** Predictors associated with culture proven infection confirmed and probable.

| **Variables** | **Odds-Ratio** | **95% CI** | **p-value** |
| --- | --- | --- | --- |
| ATB use upon ICU admission | 0.65 | 0.36 to 1.18 | 0.163 |
| Immunosuppression | 2.20 | 1.14 to 4.24 | 0.0180 |
| Maximum serum glucose on antimicrobial day (mg.dL^-1^/10)^a^ | 1.03 | 0.99 to 1.06 | 0.116 |

CI, Confidence Interval; ATB, Antimicrobial.

^a^ Divided for 10 to make odds ratio interpretation easier.

Hosmer-Lemeshow test with p-value < 0.001. AUC = 0.59.

**Table S3** Predictors associated with infection present (confirmed, probable or possible) dichotomized according to clinical practice.

| **Variables** | **Odds-Ratio** | **95% CI** | **p-value** |
| --- | --- | --- | --- |
| DeltaSOFA | 1.16 | 1.01 to 1.33 | 0.0313 |
| ATB use upon ICU admission | 1.79 | 0.84 to 3.78 | 0.127 |
| Maximum heart rate higher than 120 beats per minute | 0.56 | 0.27 to 1.15 | 0.113 |
| Leucocytes higher than 15.000 cells.mm^-3^ | 1.85 | 0.90 to 3.77 | 0.0914 |

CI, Confidence Interval; deltaSOFA, Variation in SOFA score in the previous 24h before antimicrobial start; ATB, Antimicrobial.

Hosmer-Lemeshow test with p-value < 0.001. AUC = 0.67.
